# Gender differences in hemispheric asymmetry for face processing

**DOI:** 10.1186/1471-2202-7-44

**Published:** 2006-06-08

**Authors:** Alice M Proverbio, Valentina Brignone, Silvia Matarazzo, Marzia Del Zotto, Alberto Zani

**Affiliations:** 1 Department of Psychology, University of Milano-Bicocca, Viale dell'Innovazione 10, 20126, Milan, Italy; 2 Institute of Bioimaging and Molecular Physiology (CNR), Via Fratelli Cervi 93, 20090, Milano-Segrate, Italy

## Abstract

**Background:**

Current cognitive neuroscience models predict a right-hemispheric dominance for face processing in humans. However, neuroimaging and electromagnetic data in the literature provide conflicting evidence of a right-sided brain asymmetry for decoding the structural properties of faces. The purpose of this study was to investigate whether this inconsistency might be due to gender differences in hemispheric asymmetry.

**Results:**

In this study, event-related brain potentials (ERPs) were recorded in 40 healthy, strictly right-handed individuals (20 women and 20 men) while they observed infants' faces expressing a variety of emotions. Early face-sensitive P1 and N1 responses to neutral vs. affective expressions were measured over the occipital/temporal cortices, and the responses were analyzed according to viewer gender. Along with a strong right hemispheric dominance for men, the results showed a lack of asymmetry for face processing in the amplitude of the occipito-temporal N1 response in women to both neutral and affective faces.

**Conclusion:**

Men showed an asymmetric functioning of visual cortex while decoding faces and expressions, whereas women showed a more bilateral functioning. These results indicate the importance of gender effects in the lateralization of the occipito-temporal response during the processing of face identity, structure, familiarity, or affective content.

## Background

Using functional magnetic resonance imaging (fMRI), Kanwisher and coworkers [1] found an area in the fusiform gyrus that was significantly more active when the subjects viewed faces than when they viewed assorted common objects. The authors concluded that this area, hereafter called the fusiform face area (FFA), might be specifically involved in the perception of faces, not ruling out that other structures might be play a role in this process.

Indeed, Haxby et al. (2000) provided evidence that face perception involves a distributed and hierarchically organized network of the occipito-temporal regions. In this model, the core system consists of the extrastriate visual cortex (FFA), which mediates the analysis of face structure, while the superior temporal sulcus (STS) mediates the analysis of changeable aspects of the face, such as eye gaze, facial expression, and lip movements.

Interestingly, the Kanwisher et al. study [1] showed an activation of FFA only in the right hemisphere in about half the subjects (both men and women), whereas the other subjects showed bilateral activation. These results raised the possibility of functional hemispheric asymmetry in the FFA. Studies addressing this possibility have provided conflicting evidence: several human [2-6] and animal studies [7] found stronger activity in the right hemisphere, while other studies failed to support the notion of a strict right-lateralization (e.g. [8] performed in 5 men and 7 women).

Closer examination of several studies offers more details, but no consensus, on hemispheric asymmetry in areas devoted to face processing. Yovel and Kanwisher [9] found significantly higher fMRI responses to faces than to objects in both the left and right mid-fusiform gyrus regions, although this effect was slightly greater in the right than the left FFA. In another fMRI study [10], a region that responded more strongly to faces than to objects was found within the right fusiform gyrus in 8 subjects (both women and men); however, in 6 of these subjects the same significant pattern was also found in the left fusiform gyrus. Recently, Pourtois and coworkers [11] performed an fMRI study on face identity processing on a group of 8 men and 6 women. Results revealed a reduced response in the lateral occipital and fusiform cortex with face repetition. Specifically, view-sensitive repetition effects were found in both the left and right fusiform cortices, while the left (but not right) fusiform cortex showed viewpoint-independent repetition effects. These findings were interpreted as a sign of left hemisphere dominance in terms of the ability to link visual facial appearance with specific identity knowledge. In line with this, a case has been reported of hyperfamiliarity for unknown faces after left lateral temporo-occipital damage in a female patient [12], suggesting a possible role of the left hemisphere in identity processing. Again, a recent fMRI study [13] carried out on 8 women and 7 men provided evidence of a significant activation of right fusiform and occipital gyrus (2260 voxels), left fusiform gyrus, left inferior, and middle temporal gyrus (3022 voxels) for a face familiarity effect during gender classification, thus providing a complex lateralization pattern for processing face structures and properties.

Event-related potential (ERP) and magnetoencephalography (MEG) recordings of brain activity have provided crucial information about the temporal unfolding of neural mechanisms involved in face processing (see a list of recent papers in Table 1). In particular, these recordings have identified a posterior-lateral negative peak at a latency of approximately 170 ms (referred to as "N170"). This peak has a larger amplitude in response to faces than to other control stimuli (such as houses, objects, trees, or words), and is sensitive to face inversion (upright vs. inverted). N170 is thought to reflect processes involved in the structural encoding of faces. In addition, several studies have found that affective information modulates brain response to human faces as early as 120–150 ms [14-17]. The combination of electromagnetic and functional neuroimaging data identified the possible generator of N1 in the ventral occipito-temporal cortex (FFA and superior temporal sulcus or STS) [16,18,19,53], suggesting that N1 might be the electromagnetic manifestation of a face-processing area activity. An analysis of the relevant literature shows that the topographic distribution of the face-specific N170 is not always right-sided in right-handed individuals. Based on a thorough review of methods and subject samples used in the relevant literature (see Table 1), we hypothesized that this topographic distribution might depend on marked inter-individual differences, possibly related to viewer gender.

It is of great interest to note that face-specific N170 responses were found to be bilateral or even left-sided in studies involving a sample in which women were the majority [20-24]. Equally interesting, in a recent paper on prosopagnosia in which both male and female patients were considered [20], 2 out of the 3 male patients showed an M170 response which was not sensitive to faces (as opposed to houses) while the third patient showed a right-sided sensitivity to faces. As for the two female patients, one of them showed a lack of sensitivity to faces at M170 level, while the second one showed a left-sided sensitivity.

Many face processing studies using MEG, ERP, neuroimaging, or neuropsychological data do not take subject gender into account as a variable that might affect asymmetry in brain activation. The specific goal of this study was to investigate the timing and topography of brain activity in men and women during processing of neutral and affective faces in order to detect whether there are gender differences in lateralization. To address this question, early face-sensitive P1 and N1 responses over the occipital/temporal cortices to neutral and affective expressions were measured in strictly right-handed men and women.

## Results

### Behavioral data

A repeated-measures ANOVA was performed on mean response times (RTs), but showed no significant gender effect on response speed (F[1,38] = 0.617; *p *= 0.44; females = 658 ms, males = 672 ms). Men and women subjects also did not significantly differ in accuracy; however, error percentages were too few to be statistically analyzed. The emotional valence of faces affected RTs (F[1,38] = 191; *p *< 0.000001), which were faster to negative expressions (613 ms) than to neutral expressions (717 ms) for all viewers.

### Latency

**Figure 1:**
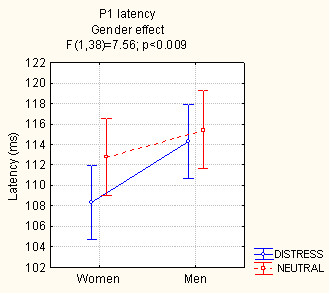
Mean latency (in ms) of the P1 component recorded at the lateral occipital area (independent of hemispheric site) and analyzed according to subject gender and type of facial expression.

Overall, P1 was earlier in response to distressed (111 ms, SE = 1.26) vs. neutral faces (114 ms, SE = 1.33), as shown by the significant "emotion" factor (*F*[1,38] = 8.65, *p *< 0.005). The analysis of P1 latency values also showed a strong "gender" effect (*F*[1,38] = 7.56; *p *< 0.009) with earlier P1 responses in women (111 ms, SE = 1.11) than men (115 ms, SE = 1.11), as shown in Fig. 1.

**Figure 2:**
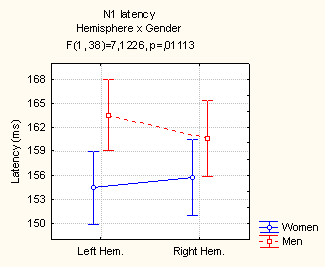
Mean latency (in ms) of the N1 component recorded at the left and right lateral occipital areas and analyzed according to subject gender.

The ANOVA performed on N1 latency values showed that responses to both neutral and distressed faces were significantly faster in women (155.1 ms, SE = 1) than men (162.1 ms, SE = 1) as shown by the significant "gender" factor (*F*[1,38] = 24.40; *p *< 0.000001). Furthermore, the effect of "hemisphere X gender" (*F*[1,38] = 7.12; *p *< 0.01) proved a strong hemispheric asymmetry in men but not in women; men responded earlier in the right hemisphere rather than the left (see Fig. 2), as confirmed by Tukey post-hoc comparisons.

### Amplitude

**Figure 3:**
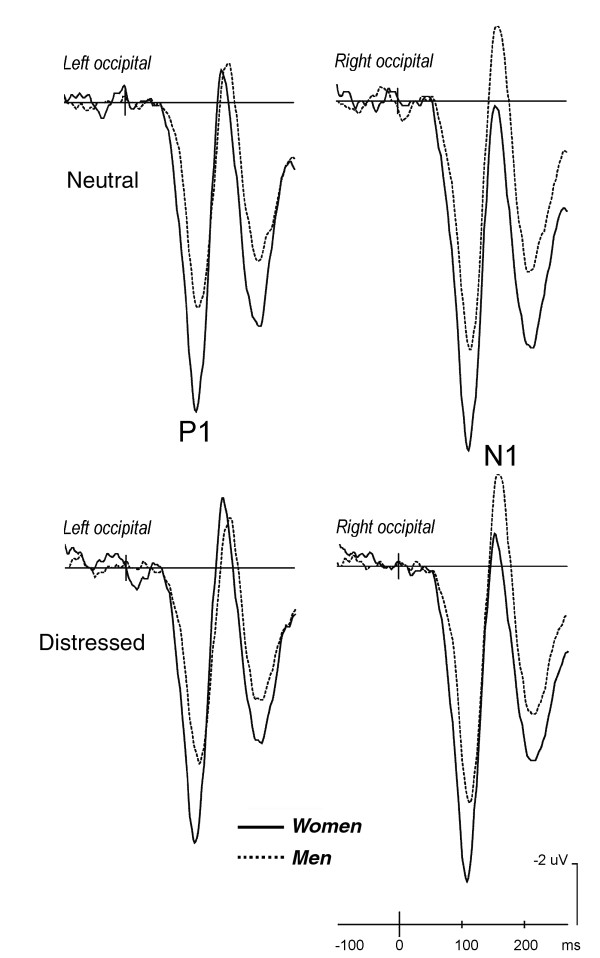
Grand-average ERPs recorded at left and right occipital sites in response to neutral and affective faces according to subject gender (women = solid line, men = dashed line).

The P110 response was much larger in amplitude in women (7.9 μV, SE = 0.79) than men (10.9 μV, SE = 0.79) as confirmed by the "gender" factor (*F*[1,38] = 7.11; *p *< 0.01), regardless of facial expression. The P1 response reached its maximum amplitude over the right occipital cortex in both genders (*F*[1,38] = 9.72; *p *< 0.0035) and was not sensitive to the affective content of the images. These effects are clearly visible in the ERP waveforms displayed in Fig. 3.

The emotional content of facial expressions significantly affected N1 amplitudes, as proved by the significance of "emotion" factor (*F*[1,38] = 6.91; *p *< 0.015) indicating larger N1 responses to distressed faces (-3.22 μV, SE = 1.1) than to neutral faces (-2.67 μV, SE = 0.84).

**Figure 4:**
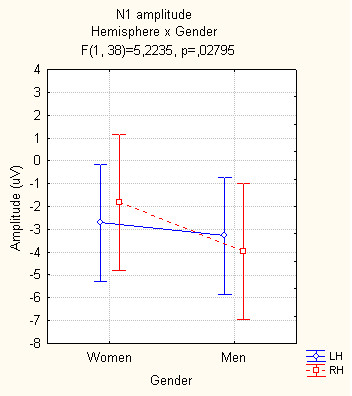
Mean amplitude (in μV) of the N1 component recorded at left and right lateral occipital areas and analyzed according to subject gender.

The N160 response was differently lateralized in men and women. Overall (and irrespective of facial expression), women exhibited a N1 response of comparable amplitude over the two visual areas (with a tendency to be larger over the LH), whereas N1 was significantly lateralized over the right hemisphere in men (see Fig. 4) as demonstrated by the significant interaction of "gender X hemisphere" (*F*[1,38] = 5.22; *p *< 0.03). This suggests a functional characterization of the hemispheric lateralization in men, which would be more related to the analysis of structural properties of faces and expressions rather than to their affective content.

## Discussion

**Figure 5:**
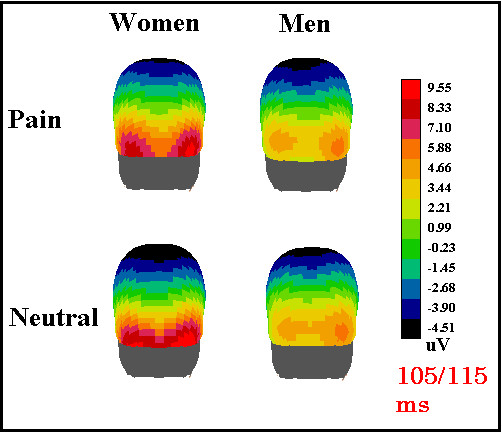
Back view of the scalp distribution of surface potentials recorded in the latency range of P110 according to subject gender and type of facial expression.

The P1 response was larger and earlier in women than in men, probably suggesting a female preference for the visual signal (infants' faces). This hypothesis is supported by a recent fMRI study showing a stronger activation of the fusiform gyrus in women (compared to men) in response to children's faces [25]. In our study, both P1 and N1 were affected by the emotional content of faces, being earlier (P110) and larger (N160) in response to distressed faces as opposed to neutral faces. These data fit with the available literature which supports the notion of early effects of emotional [14-17] and attentional factors [26-28] in the first stages of visual cortical processing. Overall, the P1 component was larger over the right occipital area in all individuals, and to all stimuli, as clearly visible from the topographic maps in Fig. 5.

This effect might be due either to sensory or cognitive factors. Since all stimuli were faces, in this experiment the asymmetry cannot be ascribed to a generic effect of face processing. Indeed, the literature on face recognition does not support the evidence of a right lateralization for the P1 response, but, rather, a bilateral distribution is often reported (when P1 is considered, see Table 1). Furthermore, in studies involving visual-spatial or selective attention tasks, the P1 component is often described as larger at the right than the left occipital lateral sites both for space orienting (e.g., [29]) and processing of global configurations (e.g., [30]). In addition, P1 is always right-lateralized in response to low spatial frequency patterns even in passive viewing conditions [31,32]. For these reasons, we cannot discuss the P1 right lateralization as an index of a hemispheric dominance for face processing.

**Figure 6:**
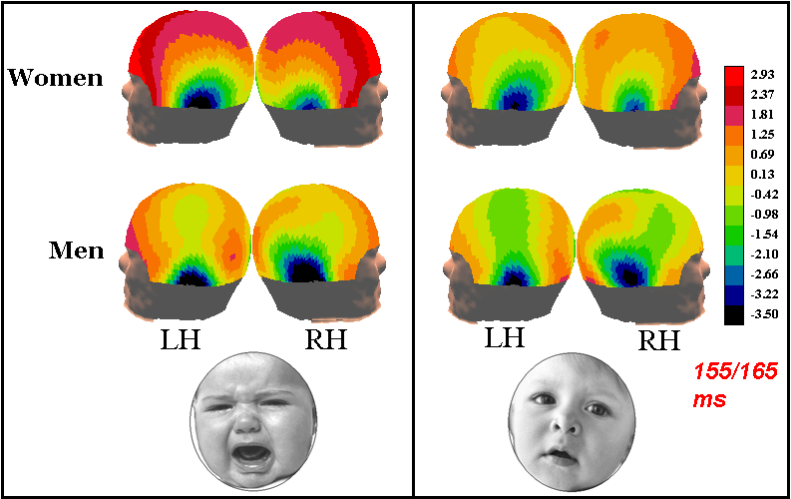
Lateral views of the scalp distribution of surface potentials recorded in the latency range of N160 according to subject gender and type of facial expression.

On the other hand, the face-specific N160 component was clearly lateralized differently in the men and women in our study. Indeed, a strong gender effect in the hemispheric lateralization of N1 component was observed, both in the latency and amplitude of cerebral response. This hemispheric asymmetry in men was not restricted to the processing of affective faces, and was significant in response to both neutral and distressed faces (see topographic maps in Fig. 6, displaying N1 scalp voltage distribution). Thus, a right hemispheric dominance is suggested for face processing in men but not in women. This may explain the many inconsistencies present in the relevant ERP and neuroimaging literature, which sometimes predicts a bilateral effect and other times a strong right-sided activity in regions devoted to face processing. These conclusions often rely on a mixed gender population, in which men and women are not necessarily equally represented (see Table 1).

Our results are also in line with many studies that show gender differences in the degree of lateralization of cognitive and affective processes. Considerable data support greater hemispheric lateralization in men than women for linguistic tasks [33] and for spatial tasks [34]. Gender differences have also been found in the lateralization of visual-spatial processes such as mental rotation [35] and object construction tasks [36], in which males are typically right hemisphere (RH) dominant and females bilaterally distributed. More relevant to the present experiment are the data provided by Bourne [37], who examined the lateralization of processing positive facial emotion in a group of 276 right-handed individuals (138 males, 138 females). Subjects were asked to observe a series of chimeric faces formed with one half showing a neutral expression and the other half showing a positive expression in the left or right visual field, and to decide which face they thought looked happier. The results showed that males were more strongly lateralized than women in the perception of facial expressions, showing a stronger perceptual asymmetry in favour of the left visual field. There are also a number of studies that have found different degrees of lateralization in the cerebral response of men and women to emotional stimuli [38-41]: men tend to demonstrate an asymmetric functioning, and women a bilateral functioning [42].

## Conclusion

Our study found a lesser degree of lateralization of brain functions related to face and expression processing in women than men. Furthermore, these results emphasize the importance of considering gender as a factor in the study of brain lateralization during face processing. In this light, our data may also provide an explanation of the inconsistencies in the available literature concerning the asymmetric activity of left and right occipito-temporal cortices devoted to face perception during processing of face identity, structure, familiarity or affective content.

## Methods

### Participants

40 healthy individuals (20 women and 20 men) with normal or corrected-to-normal vision volunteered for this study. All participants were strictly right-handed as assessed by the Edinburgh Inventory [43] and had a strong right-eye dominance (as attested by practical tests, such as looking inside a bottle or alternately closing each eye to evaluate parallax entity). They were of similar age (average = 33.7 years) and socio-cultural status. Experiments were conducted with the understanding and the written consent of each participant and in accordance with ethical standards (Helsinki, 1964). The study was approved by the CNR Ethical Committee.

### Materials and procedures

Participants sat about 120 cm from a computer monitor in an acoustically and electrically shielded cabin. They were instructed to focus on a small cross located in the centre of the screen and to avoid any body or eye movements. Stimuli were randomly presented in the centre of the screen for about 900 ms with an ISI of 1300–1900 ms. The stimulus set consisted of 160 high resolution black and white photos of infants expressing neutral or affective (distressed) emotional states. The electroencephalogram (EEG) was continuously recorded and synchronized with the onset of picture presentation. The task consisted of deciding on the emotional content of the picture. The responses were to be made as accurately and quickly as possible by pressing a response key with the right index finger of the right or left hand (to signal distress or well-being). The hand and experimental run orders were counterbalanced across subjects.

The EEG was continuously recorded from 28 scalp electrodes mounted on an elastic cap. The electrodes were located at frontal (Fp1, Fp2, FZ, F3, F4, F7, F8), central (CZ, C3, C4), temporal (T3, T4), posterior-temporal (T5, T6), parietal (PZ, P3, P4), and occipital scalp sites (OZ, O1, O2) of the International 10–20 System. Additional electrodes were placed halfway between anterior-temporal and central sites (FTC1, FTC2), central and parietal sites (CP1, CP2), anterior-temporal and parietal sites (TCP1, TCP2), and posterior-temporal and occipital sites (OL, OR). Vertical eye movements were recorded using two electrodes placed below and above the right eye, while horizontal movements were recorded from electrodes placed at the outer canthi of the eyes. Linked ears served as the reference lead. The EEG and the EOG were amplified with a half-amplitude band pass of 0.01–70 Hz. Electrode impedance was kept below 5 kΩ. Continuous EEG and EOG were digitized at a rate of 512 samples per second.

Computerized rejection of artefacts was performed before averaging to discard epochs in which eye movements, blinks, excessive muscle potentials, or amplifier blocking occurred. The artefact's rejection criterion was a peak-to-peak amplitude exceeding ± 50 μV and the rejection rate was about 5%. ERPs were averaged offline from 100 ms before until 1000 ms after presentation of the final word. ERP trials associated with an incorrect behavioural response were excluded from further analysis. For each subject, distinct ERP averages were obtained according to infant's facial expression. ERP components were identified and measured with reference to the baseline voltage averages over the interval from -100 ms to 0 ms. P1 and N1 peak amplitude and latency values were measured at lateral occipital sites (OL, OR), where both components reached their maximum amplitude, in the time window between 90–140 ms and 145–175 ms. The McCarthy-Wood correction, sometimes used to normalize ERP amplitudes, was not applied to our data, in line with recent findings in the literature [60].

ERP data of amplitude and latency were analyzed by means of 3- and 4-way repeated measure ANOVAs; the P1 and N1 component were analyzed separately. For P1, there was one between-group factor, "gender" (women and men) and two within-group factors, "emotion" (neutral, distress) and "cerebral hemisphere" (left and right). For N1 there was the extra within-group factor "electrode site" (lateral/occipital, posterior/temporal). Behavioural data were analyzed by means of a 2 way repeated-measures ANOVA performed on mean RTs according to "gender" of viewers and emotional valence of stimuli ("emotion" factor).

## Authors' contributions

AMP conceived of the study, coordinated data acquisition and analysis, interpreted the data and drafted the manuscript. SM, VB and MDZ (funded by Fondazione San Raffaele del Monte Tabor) participated in the design of the study, carried out data collection and performed statistical analyses. AZ participated in the study design and coordination and helped to draft the manuscript. All authors read and approved the final manuscript.

## Figures and Tables

**Table 1:** Recent ERP and MEG papers reporting P1 and N170 topographic distributions. ERP components were recorded in response to faces and other visual objects over the left and right occipital/temporal areas.

**Papers (Authors and year)**	**# Ss**	**Gender**		**P1 and N1 distribution and effects**	**Right asymmetry for face processing**	
		**F**	**M**		**P1**	**N1**

[14] Batty & Taylor (2003)	26	13	13	P1 larger over the RH but not sensitive to emotions or face-specific. N1 emotion-specific but bilateral.	Yes (Not face- specific)	No
[43] Bentin et al. (1999)	1	-	1	Prosopagnosic patient with right temporal abnormality and not face-sensitive N170.		Yes
[44] Caldara et al. (2004)	12	6	6	N1 larger over the RH to both Asian and Caucasian faces.	-	Yes (not race-specific)
[45] Campanella et al. (2000)	12	-	12	Face identity sensitive N170 larger at right posterior/temporal site.	-	Yes
[46] Esslen et al. (2004)	17	10	7	N170 to neutral faces activates the right fusiform gyrus (LORETA).	-	Yes
[47] George et al. (2005)	13	7	6	Mooney faces. P1 bilateral not modulated by face inversion. N1 larger on the right hemisphere to both inverted and upright faces.	No	Yes (to both inverted and upright faces)
[48] Gliga et al. (2005)	10	7	3	N1 larger over the RH to both bodies and faces.	-	Yes
[15] Halgren et al. (2000)	10	2	8	Overall, laterality greater on the right than the left in fusiform face-selective activity, but a high level of individual variability.	No	Yes
[20] Harris et al. (2005)	2	2	-	2 female prosopagnosic patients. For NM M170 not face-sensitive. Face selectivity effect (faces vs. houses) for KL >LH (RH = 17.3 fT, LH = 26.9 fT).	-	No
[20] Harris et al. (2005)	3	-	3	3 male prosopagnosic patients. For EB and KNL M170 not face-sensitive. Face selectivity effect for ML > RH. (RH = 57.6 fT, LH = 35.6 fT).	-	Yes
[20] Harris et al. (2005)	17	9	8	M170 larger to faces than houses and tended to be larger at LH (*p *< 0.08).		No
[18] Henson et al. (2003)	18	8	10	Face sensitive N170 was larger at superior temporal area.	-	Yes
[49] Herrmann et al. (2005)	39	19	20	Bilateral P1 and N1 larger to faces than buildings.	No	No
[50] Holmes et al. (2005)	14	5	9	Not specifically mentioned. From inspection of Fig. 2. P1 much larger on the RH to both faces and houses. N1 larger on the RH to faces only (unfiltered stimuli).	Yes (Not face- specific)	Yes
[51] Itier & Taylor (2004)	36	18	18	Face specific P1 was bilateral. N170 was larger over the RH at parietal/occipital sites.	No	Yes
[52] Itier & Taylor (2004)	16	7	9	N170 to upright faces is bilateral	-	No
[53] Itier & Taylor (2004)	16	7	9	Face-specific P1 is larger over the RH. N1 was bilateral at occipital sites. It was larger at right parietal sites to objects, inverted faces, and upright faces.	Yes	Yes/no (to objects, inverted and upright faces.)
[21] Jemel et al. (2005)	15	10	5	No hemispheric asymmetry for P1 or N1 to faces.	No	No
[54] Kovacs et al. (2005)	12	4	8	Face (vs. hand)-specific N170 was larger over the RH.	-	Yes
[55] Latinus et al. (2005)	26	13	13	Mooney faces. Bilateral or left-sided P1 not sensitive to face-inversion. N1 larger on the RH to upright faces.	No	Yes
[19] Liu et al. (2000)	17	*	*	M170 larger to faces than animal and human forms at bilateral occipital/temporal sensors.	-	No
[22] Meeren et al. (2005)	12	9	3	Face-body compound images: lead main effect (*p *= 0.04) for P1 amplitude with O1>O2>Oz, but post hoc tests failed to reveal significant differences. ERPs to isolated faces: P1 and N1 to angry and fearful faces were not right-sided.	No	No
[16] Pizzagalli et al. (2002)	18	7	11	N1 larger over the right fusiform gyrus and affected by face likeness.	-	Yes
[17] Pourtois et al. (2005)	13	9	4	Unfiltered faces: P1 affected by emotional content (fear vs. neutral) in both hemispheres. N1 strongly right-lateralized to upright vs. inverted faces. LAURA source estimation for P1 and N1 topography in the left extra-striate visual cortex	No	Yes (but LH generator for source estimation)
[23] Righart & Gelder (2005)	12	10	2	N170 amplitudes were more negative for faces in fearful contexts compared to faces in neutral contexts, but only significantly for electrodes in the left hemisphere.	No	No
[56] Rossion et al. (1999)	14	5	9	N1 larger at posterior temporal sites to inverted faces.	No	Yes (not upright specific)
[6] Rossion et al. (2003)	16	6	10	N170 for faces compared to words in the right hemisphere only.	No	Yes
[57] Rousselet et al. (2004)	24	12	12	P1 larger on the RH for both objects, animal and human faces. N1 much larger on the RH for face than objects, but asymmetry found for objects as well.	Yes (Not face- specific)	Yes/No (Not face- specific)
[24] Valkonen-Korhonen et al. (2005)	19	15	4	Control group: N1 Larger at T5/T6 in an emotion detection task (happy upright faces).	-	No
[58] Yovel et al. (2003)	12	7	5	N1 to symmetrical and left or right hemi-faces was larger at right temporal site.	-	Yes
